# Looking at the upper facial half enlarges the range of holistic face processing

**DOI:** 10.1038/s41598-023-29583-z

**Published:** 2023-02-10

**Authors:** Zhe Wang, Hao Ni, Xin Zhou, Xiteng Yang, Ziyi Zheng, Yu-Hao P. Sun, Xiaohui Zhang, Haiyang Jin

**Affiliations:** 1 Department of Psychology, Zhejiang Sci-Tech University, Zhejiang, China; 2 Division of Science, Department of Psychology, New York University Abu Dhabi, Abu Dhabi, United Arab Emirates

**Keywords:** Neuroscience, Psychology

## Abstract

Previous studies suggested that upper and lower facial halves might be involved in the human holistic face processing differently. In this study, we replicated and extended the finding above. In Experiment 1, we used the standard composite-face task to measure holistic face processing when participants made judgements on the upper and lower facial halves separately. Results showed that the composite-face effect was stronger for the upper facial half compared to the lower half. In Experiment 2, we investigated how facial information was integrated when participants focused on different features, using the perceptual field paradigm. Results showed that: (1) more “peripheral faces” were chosen when participants fixated at the eyes than when they fixated at the mouth; (2) less “peripheral faces” were chosen for inverted faces regardless of the fixated features. Findings from both experiments together indicate that more peripheral facial information were integrated when participants focused on the upper facial half, highlighting the significance of focusing on the upper facial half in face processing.

## Introduction

Face is a multi-dimensional complex visual stimulus. Although the processing of faces is complicated, people perceive and recognize faces precisely and efficiently. To explain this phenomenon, the holistic face processing hypothesis proposes that the perception system integrates multi-dimensional information of a face into a whole or a gestalt to process faces quickly and accurately^1^. Three classic paradigms measuring holistic face processing have provided some evidence, suggesting that there is a face-specific information integration mechanism. For example, Yin found that the inversion would significantly impair the recognition of faces, comparing to non-face objects (the face inversion effect^2,3^); Young and colleagues found that recognizing the upper half of a face would be affected by its lower half (the composite face effect^4,5^); Tanaka and colleagues found that the performance of recognizing eyes, nose or mouth was better if they were presented in the whole face than in isolation for upright faces, but not for inverted faces (the part/whole effect^6,7^). All these studies support the holistic face processing hypothesis. However, little is known about the scope of holistic processing. In the current study, we compared the roles of the upper-half face and the lower-half face involved in human holistic face processing.

It is well accepted that holistic face processing is highly selective: some facial information is more important than others. But it is not clear which dimension or variable contributes more to face perception. In early studies, a lot of evidence suggested that the configural information of faces is more important for holistic processing than featural information^8^. However, this conclusion was undermined, as later some evidence suggests that the featural and configural information is playing similar roles in face perception^9–13^.

By contrast, some studies suggest that the facial region is a critical factor in face recognition. Specifically, the information on the upper facial half may play a more important role in face recognition than the lower half^10,14–18^. For instance, Hsiao and colleagues used the eye-tracking technique and observed that the number of fixations on eyes was significantly higher than that on mouth, showing a bias fixating on the upper half^15^; Burton and colleagues used the visual search paradigm and found that participants were faster and more accurate in searching for an upper facial half than a lower half in a complex visual background^16^; Wang and colleagues using the face matching task found that participants were more sensitive to changes in the eye region relative to the mouth region^10^. In addition, some studies found that N170 amplitudes were stronger when participants fixed on eyes relative to other regions^19,20^. Considering that the N170 is regarded as the reflection of ERP index of holistic face processing^21,22^, this may suggest that the processing of the upper half of the face (than the lower half of the face) is more involved in holistic face processing.

This study aims to investigate whether the range of holistic face processing varies when people fixate on different facial regions (e.g., the upper half and the lower half). We hypothesized that the range of holistic face processing should be relatively larger when participants fixated on the upper half than the lower half. This study includes two experiments where different experimental paradigms were used. These two experiments had one thing in common: both predefined a facial area as the center of gaze and measured the holistic face processing range.

Experiment 1 used the standard composite-face task^4,23^, in which each trial presented two faces successively, and participants were instructed to judge whether the upper halves (or the lower halves, block design) of two faces were same or not (see "Experiment 1" "Method" part for details). The range of holistic face processing in the composite-face paradigm was operationally defined as the influence of irrelevant facial halves on the target halves. We expected to observe that participants would be more interfered by the irrelevant facial half when judging the upper relative to the lower facial half.

Experiment 2 used the perceptual field paradigm developed by Van Belle and colleagues^24^. On each trial, a combined face with the fixated part from a face ("the central face") and the remaining part from another face ("the peripheral face") is presented, and then the two original faces are presented simultaneously. Participants were instructed to determine which original face was more similar to the combined face (see “Experiment 2” “Method” part for details). In the perceptual field paradigm, the percentage of choosing "peripheral faces" reflected the holistic face processing range. We expected that participants would have higher percentage of choosing "peripheral faces” when the eyes (the left eye or the right eye) located at the foveal area relative to when the mouth located at the foveal area.

## Experiment 1

### Methods

#### Participants

We used G-Power 3.1^25^ to plan the sample size. For a 2*2 within-subject design experiment, a power analysis indicated that a sample size of 30 be required to detect medium effect size (0.25) at the 0.05 alpha level with 0.9 power value.

Thirty undergraduates (12 males, mean age = 18.8 ± 0.8 years) were recruited. All participants had normal or corrected-to-normal vision, were right-handed based on self-report and were paid for participation. All participants provided written informed consent. All methods and procedures used in this study conformed to ethical guidelines for testing human participants. Specific written informed consent has been obtained to publish the images in an online open-access publication. The study was approved by the Human Research Ethics Committee of Zhejiang Sci-Tech University (decision: 201409P01).

#### Stimuli

Twenty Chinese full-front faces (10 female) with neutral expression were used. There was no jewelry, glasses, or makeup on these faces. The photographs were converted to gray-scale (256 × 256 pixels). An oval contour was used to remove all the external cues (hairstyle, ears, accessories, head shape).

We split all faces into the upper and lower halves from the noses and re-combined the upper halves with a randomly assigned lower half from another face to make 20 aligned and 20 misaligned composite faces. So one composite face shared the same upper half with one original face and the same lower half with another original face.

**Figure 1:**
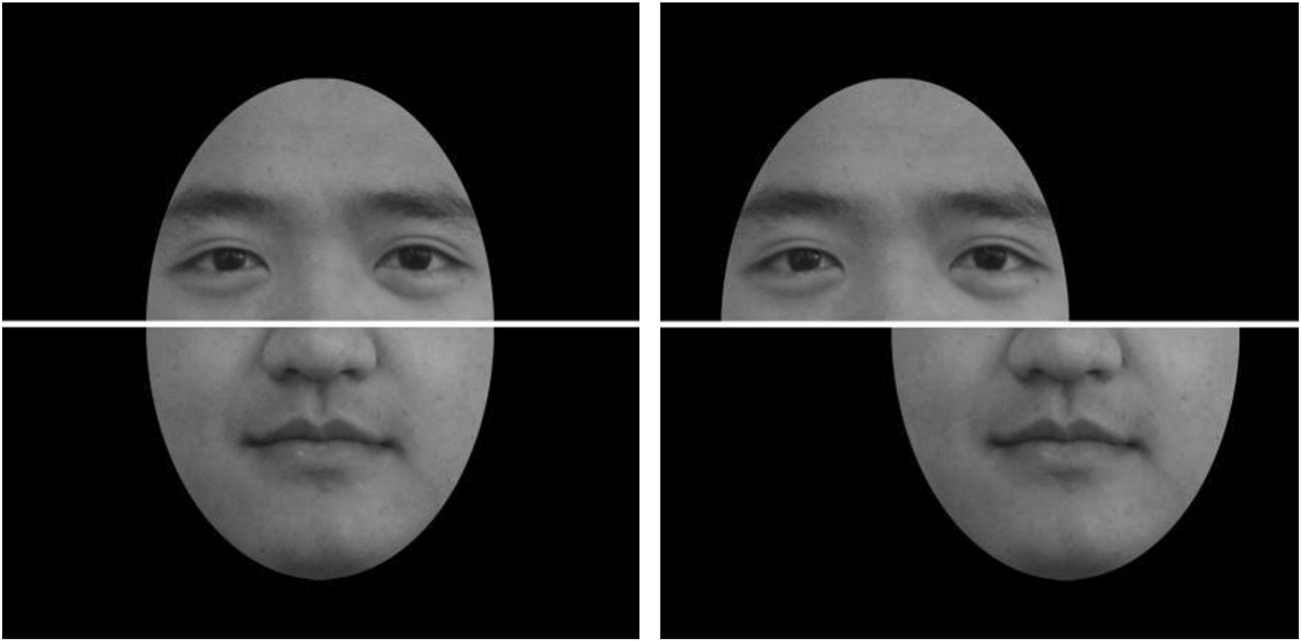
Samples of aligned (left) and misaligned (right) faces.

A 3 pixels white line was added to the original faces and their composite faces to make it clear to participants where were the upper and lower halves. Misaligned faces were created by offsetting the two halves to opposite directions by 70 pixels (Fig. 1).

We combined all faces into three trial types: (i) the upper halves of the study and test faces were the same, but the lower halves were different; (ii) the lower halves of the study and test faces were the same, but the upper halves were different; (iii) both halves of study and test faces were different.

#### Procedure and design

The experiment was administrated by E-prime 2.0 in a quiet room with a 17-inch CRT screen (refresh rate: 85 Hz, resolution: 1024 × 768 pixels).

**Figure 2:**
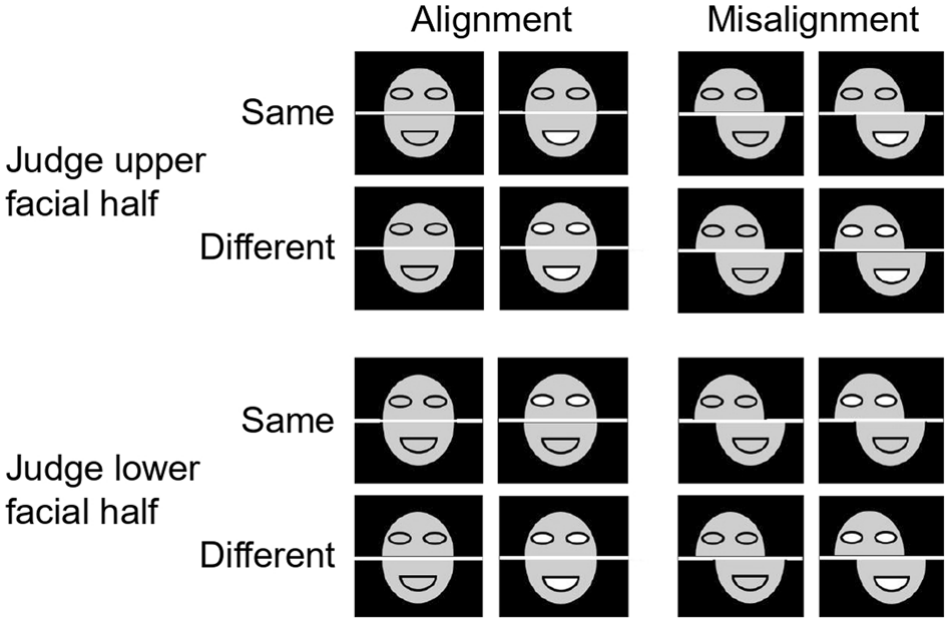
Schematic diagram of experiment 1 face matching. When judging the upper halves of the two faces, the upper halves can be the same or different, but the lower halves are always different (the top two lines); when judging the lower halves of the two faces, the lower halves can be the same or different, but the upper halves are always different (the bottom two lines).

**Figure 3:**
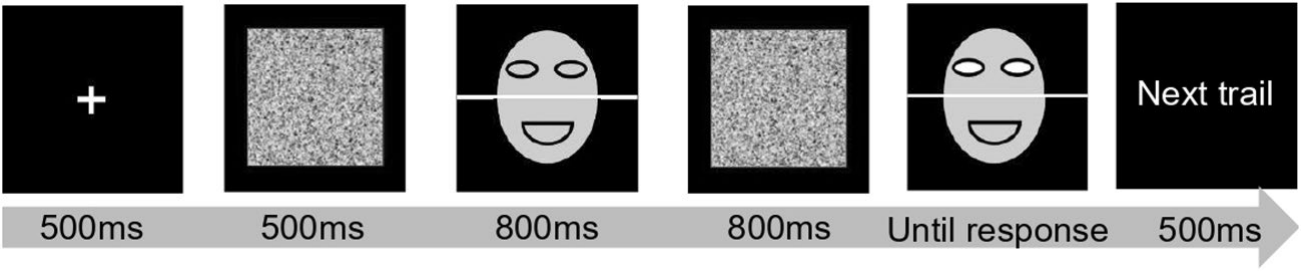
The schematic diagram of the process of Experiment 1. In this trial, the lower half is the target, and the correct response is “same”, because the lower halves of the two faces are identical.

On each trial, a fixation cross was first presented on the screen center for 500 ms. After a noise mask displayed for 500 ms, a study face was presented for 800 ms. This was followed by the noise mask for 500 ms. Then a test face showed up and would not disappear until a response was collected. Participants were asked to press Key "1" or "2" to indicate whether the target half of the study face was the same as that of the test face. The keys were counterbalanced among participants. On the "same" trials, either the upper or the lower halves of the two faces were the same; On the "different" trials, both the upper and lower halves of the two faces were different (Fig. 2). To avoid participants responding by matching image properties instead of facial identities, the position of target face was slightly offset. Then, "Next trial" was display for 500 ms to indicate the next trial. The procedure of a trial is shown in Fig. 3.

Each participant made judgements on upper and lower halves in two different blocks, and the order of blocks was counterbalanced among participants. Each block included 2 (Alignment: aligned vs. misaligned) × 2 (Trial correct response: same vs. different) × 20 faces = 80 trials. The trial orders in each block were randomized. Each participant completed 160 trials in total.

Before the formal experiment, participants completed 16 practice trials. The procedure of the practice trials was the same as the formal experiment, except that the stimuli were line-drawing face images, and feedback was provided for each practice trial.

### Results and discussion

Following previous literature using the standard composite face task^4,23^, we only analyzed the data of the same trials. We excluded trials in which the response time was less than 200 ms or outside 2 standard deviations for each participant separately. As a result, 4.9% trials on average were not included in following analysis.

**Figure 4:**
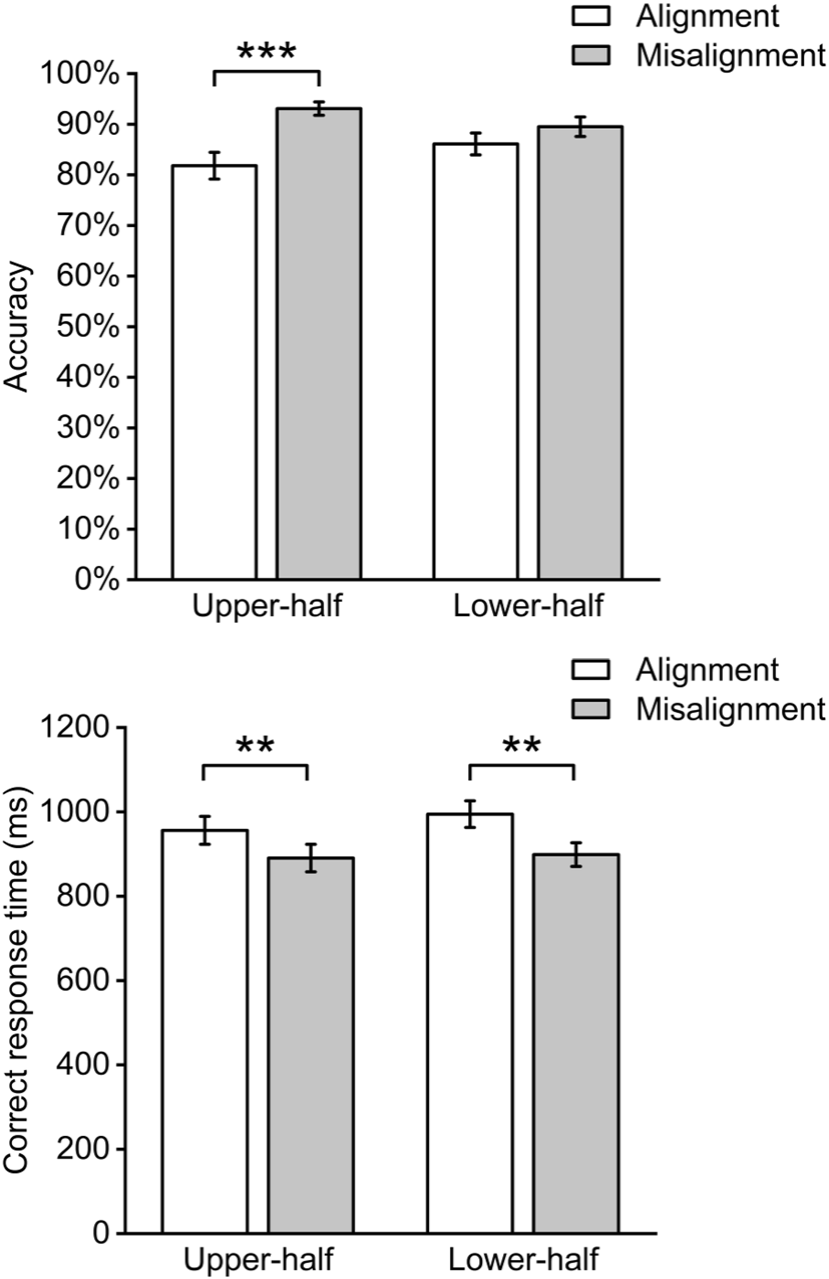
The accuracy (top) and the correct response time (bottom) of Experiment 1. Error bars indicate one standard error from the mean.

A 2 × 2 repeated-measures ANOVA was conducted on accuracy, with two within-subject factors of location (upper or lower) and alignment (aligned or misaligned). The results (Fig. 4) showed that there was a significant main effect of alignment (*F*(1,29) = 25.78, *p* < 0.001, η_p_^2^ = 0.471); the accuracy of misaligned face (*M*_*misaligned*_ = 91.3%, *SE*_*misaligned*_ = 1.3%) was higher than aligned face (*M*_*aligned*_ = 84.0%, *SE*_*aligned*_ = 2.0%), which showed the composite effect. Notably, the interaction between location and alignment was also significant (*F*(1,29) = 5.15, *p* = 0.031, η_p_^2^ = 0.151). Simple effect analyses showed that the participants performed better on the misaligned trials than on the aligned trials, when judging the upper halves of the two faces (*t*(29) = 4.68, *p* < 0.001, d = 0.858), indicating a composite-face effect for the upper facial half; by contrast, when participants judged the lower halves of the two faces, no significant differences were found between the misaligned trials and the aligned trials (*t*(29) = 1.62, *p* = 0.116). There was no significant main effect of region (*F*(1,29) = 0.05, *p* = 0.825). These results together suggest that the composite face effect was observed for the upper facial half, and, more importantly, it was larger than that for the lower facial half.

A 2 × 2 repeated ANOVA was conducted on correct response times, with two within-subject factors of location (upper or lower) and alignment (aligned or misaligned). The main effect of alignment was significant (*F*(1,29) = 31.23, *p* < 0.001, η_p_^2^ = 0.518). The correct response times of the misaligned trials (*M*_misaligned_ = 894 ms, *SE*_misaligned_ = 25) was smaller than the aligned trials (*M*_aligned_ = 975 ms, *SE*_aligned_ = 30). There was no significant main effect of location (*F*(1,29) = 0.72, *p* = 0.402, η_p_^2^ = 0.024) or interaction (*F*(1,29) = 1.95, *p* = 0.173, η_p_^2^ = 0.063).

In summary, Experiment 1 showed that holistic face processing was relatively stronger when participants fixated on the upper-half of a face, compared with the lower-half, indicating that more information is involved in face processing when participants looked at the upper facial half than the lower facial half, although this information is not relevant to the current task. This finding supports the hypothesis that the fixation location influences the range of holistic face processing, with the range of the upper facial half being larger than the lower facial half.

## Experiment 2

Rossion proposed the concept of *“perceptual field*”, which is defined as “the area of vision where the observer can extract diagnostic visual information for the task, and related terms could be the functional visual field or the perceptual spatial window”^26,27^. The perceptual field hypothesis suggests this perceptual process makes an observer see the multiple features of a whole face at once, and the perceptual process is holistic because it is driven by a holistic face representation, derived from visual experience. An inverted face cannot be perceived holistically, because the perceptual field of the observer is constricted for inverted faces, each facial feature having to be processed sequentially, independently, i.e. over a smaller spatial window than the whole face^24^. According to the definition of the perceptual field, the size of the perceptual field can serve as a reliable indicator of the range of the information used in the face holistic processing.

**Figure 5:**
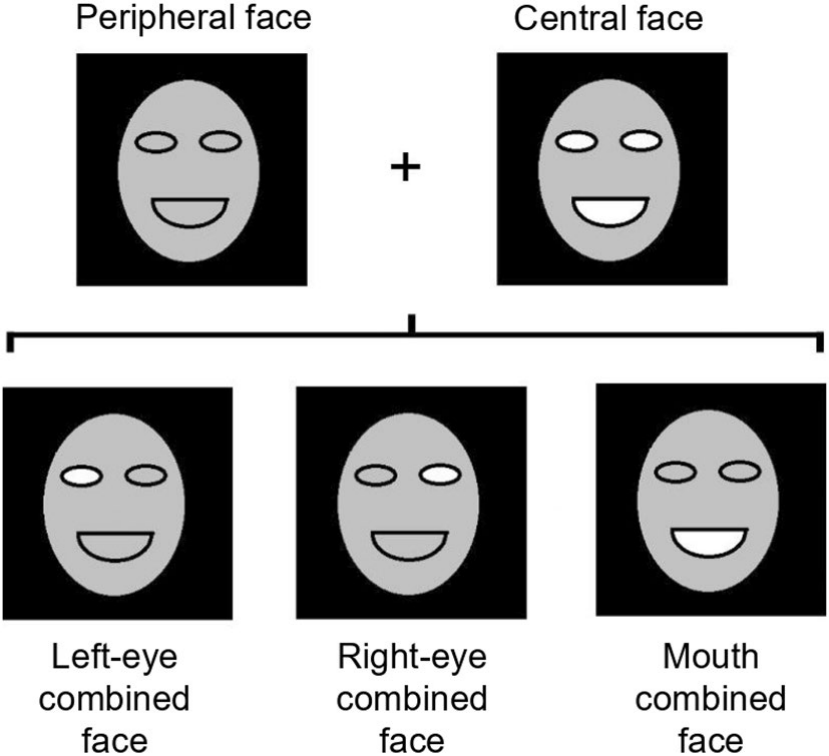
The schematic diagram of three combined faces of Experiment 2 (for every combined face, its central area comes from the central face and its peripheral area comes from the peripheral face).

Van Belle et al. developed a perceptual field paradigm using a gaze-contingent morphing approach (Fig. 5) for measuring holistic face processing^24^. The specific steps of the paradigm are: (1) After presenting a combined face, which was made of one fixated part from an original face ("the central face") and the remaining part from another original face ("the peripheral face"), the two original faces are presented simultaneously; (2) Participants are instructed to determine which original face is more similar to the combined face; (3) The percentage of choosing “peripheral faces” is the measurement of the perceptual field. The larger the percentage of choosing peripheral faces is, the larger the perceptual field  is and the stronger the holistic face processing is. The advantages of the perceptual field paradigm (compared to the composite-face task) are: (1) The stimulus is a complete face, not a split face recomposited by face halves, and thus the face perception process has high ecological validity; (2) there are no correct responses in the perceptual field paradigm, so participants should not apply flexible strategies to pursue high accuracy. Therefore, this paradigm should mitigate the confounds caused by changes in strategies. As a test of the perceptual field paradigm validity, Li and colleagues found that the perceptual field paradigm can also detect the age bias effect in face perception^28^.

Therefore, Experiment 2 used the perceptual field paradigm to measure the range of the perceptual field when participants fixated at different regions, in which the range was defined as the percentage of choosing the peripheral face. In addition, experiment 2 also set upright and inverted conditions to exclude the confusion between the spatial position (top or bottom) and the face region (eyes or mouth) in face stimuli’s spatial reference system.

According to our hypothesis, in the perceptual field paradigm we expected that: (1) The perceptual field is larger (the percentage of choosing “peripheral faces” is larger) when participants fixated at the eye region relative to the mouth region; (2) The perceptual field is dramatically reduced (the percentage of choosing “peripheral faces” is very low) when faces are inverted, no matter when participants fixate at the eye or mouth region. (3) Moreover, if the spatial position (top or bottom) rather than the face region (eyes or mouth) plays an important role in the holistic face processing, for inverted faces, the range of the perception field will be relatively larger when participants fixated at the mouth's area (at the top of the screen) than when participants fixated at the eyes' area (at the bottom of the screen).

### Methods

#### Participants

Thirty-five undergraduates (13 males, mean age = 20.9 ± 2.2 years) were recruited. All participants had normal or corrected-to-normal vision, were right-handed based on self-report and were paid for participation. Written informed consent was obtained for experimentation with human participants. All methods and procedures used in this study conformed to ethical guidelines for testing human participants. The study was approved by the Human Research Ethics Committee of Zhejiang Sci-Tech University (decision: 201409P01). For a 2*2 within-subject design experiment, a power analysis indicated that a sample size of 30 be required to detect medium effect size (0.25) at the 0.05 alpha level with 0.9 power value.

#### Materials

60 Chinese full-front colorful faces (30 females) with neutral expression were used in Experiment 2 and the size of these pictures was 408 × 408 pixels. We removed the external cues (haircut, ears, accessory, etc.) from faces, and paired faces by matching the face width and interpupillary distances (30 pairs in total), in order to minimize distortions in the combined face.

One of the two faces in each pair was used as the central face and the other was used as the peripheral face. The left eye, right eye and mouth of the central face were respectively placed on the corresponding positions in the peripheral face, forming a left-eye combined face, a right-eye combined face and a mouth combined face (Fig. 5). The overlapped area was an oval with the ratio of 1.4:1, and its width was about half of the face width. To make the combined faces natural, the margin of the components was feathered, and face skin was also adjusted slightly. All the manipulation was completed with the Adobe Photoshop CS5.

#### Procedure and design

The experiment was administrated by E-prime 2.0 in a quiet room with a 17-inch CRT screen (refresh rate: 85 Hz, resolution: 1024 × 768 pixels).

**Figure 6:**
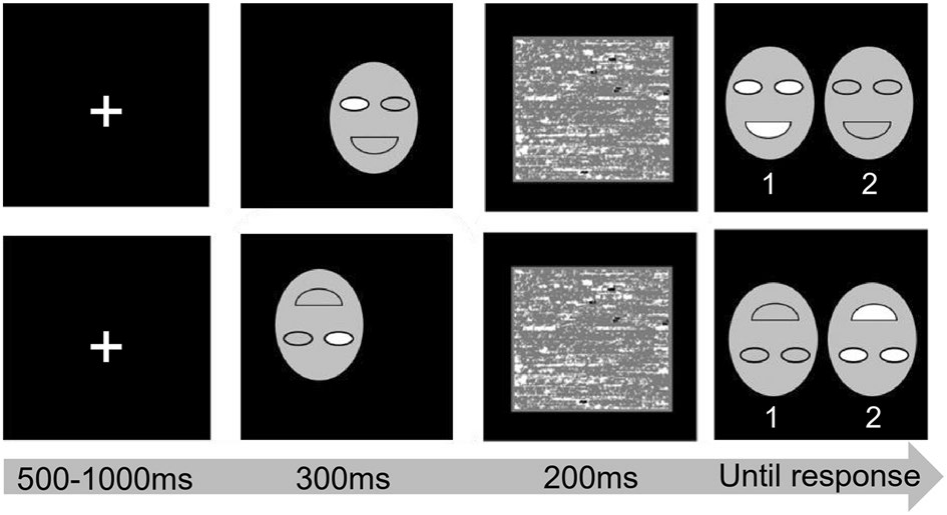
Schematic diagram of the process of Experiment 2. The first line is upright, and the second line is inverted.

On each trial, a fixation point presented on the center of screen with a random duration of 500 ms to 1000 ms; then a combined face (300 ms) was presented, followed by a mask (200 ms); after that, the two original faces were presented on the screen side by side. Participants were asked to press a key (1 or 2) to judge which face was more similar to the combined face (Fig. 6).

We used the following techniques to maintain the participants' eyes' foveas in a particular location while the combined face is shown: (1) The participants were instructed to maintain their gaze on the fixation point, and when a combined face was exhibited, its central region (the eye or mouth) was located where the fixation point had been, as shown in Fig. 6. (2) The duration time of the fixation point was random, making it impossible for participants to relocate their eyes in advance due to the anticipation of the presentation of the combined faces. (3) The participants had no opportunity to relocate their fixation away from the central region once the combined face was given since the presentation of the combined face last only 300 ms, which was close to the duration of a fixation.

**Figure 7:**
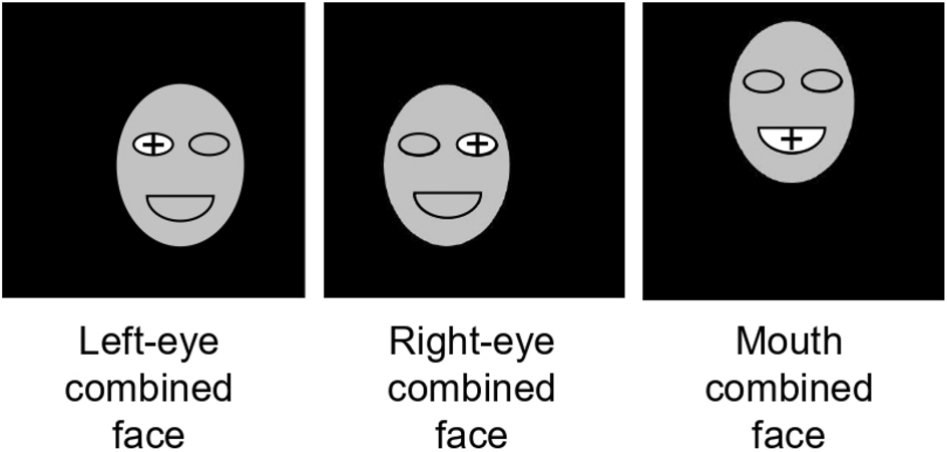
The schematic diagram of the position of the combined face in Experiment 2 (upright face).

This experiment included two within-subject variables: fixated position (left eye, right eye or mouth) and face orientation (upright or inversion). For different fixated positions (left eye, right eye or mouth), the combined faces were presented in different positions on the screen, so that their left eye, right eye or mouth could appear at the center of the screen (Fig. 7). There were not correct responses, because the combined face was made by the central face and the peripheral face. The experiment included 3 × 2 × 30 = 180 trials which were presented randomly.

Before the formal experiment, participants completed 6 practice trials. The procedure of the practice trials was the same as the formal experiment, except that the stimuli were line-drawing face images, and a feedback was provided for each practice trial.

### Result

According to the perceptual field paradigm, we analyzed the proportion that participants chose the peripheral face. We merged the data of both eyes because there was no difference between the left and right eye after comparing the results.

A 2 (fixated position: eyes or mouth) × 2 (face orientation: upright or inverted) repeated-measures analysis of variance (ANOVA) was performed, using proportion of choosing peripheral faces as the dependent variable.

**Figure 8:**
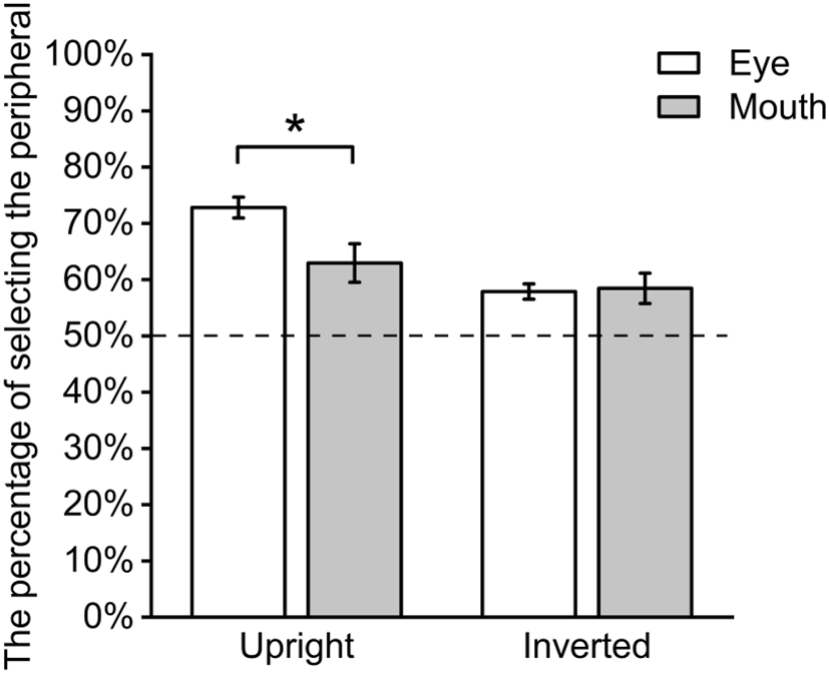
The percentage of peripheral faces chosen by participants under different conditions. Error bars indicate one standard error from the mean.

The results (Fig. 8) showed that the main effect of fixated position was not significant (*F*(1,34) = 1.96, *p* = 0.171, η_p_^2^ = 0.171), and the main effect of face orientation was significant (*F*(1,34) = 48.59, *p *< 0.001, η_p_^2^ = 0.588) , indicating that the percentage of choosing peripheral faces was higher when the faces were presented upright than the faces were presented inverted. The interaction was significant (*F*(1,34) = 15.22, *p* < 0.001, η_p_^2^ = 0.309). Simple effect analyses showed that for upright faces, the percentage of choosing peripheral faces was significantly higher when participants fixated at the eyes than the mouth (*t*(34) = 2.56, *p* = 0.015, d = 0.597), suggesting that the perceptual field was larger when participants fixated at the eyes of the upright face than fixated at the mouth. For inverted faces, the percentage difference of choosing peripheral faces between the participants fixating at the eyes and fixating at mouth was not significant (*t*(34) = − 0.19, *p* = 0.847). It seemed that perceptual fields were not different from each other, when the participants fixated at the eyes or mouth of inverted faces. Considering that the relative spatial positions of the upper-half face and lower-half face were interchanged, the experimental results had not been reversed, indicating that the relative spatial positions of the eyes or mouth did not affect the holistic face processing. Therefore, the combination of the above two results indicated that the range perceptual field covered was indeed due to changes in the gaze area (eyes or mouth) rather than the spatial position (top or bottom).

In order to estimate the holistic face processing under each condition, the percentage of choosing peripheral faces under each condition was compared with 50%. It was found that the percentage under all conditions were significantly greater than 50% (*ps* < 0.003), suggesting that the holistic face processing occurred. That indicated that the inversion did not completely damage but only severely weakened the holistic face processing^2,29–31^. At the same time, another simple effect in orientation also shows that when subjects gazing at the eyes, the percentage of choosing peripheral faces for upright face is significantly higher than for inverted face (*t*(34) = 9.32, *p* < 0.001, d = 1.561), suggesting the face inversion effect seriously damaged the perceptual field centered on eyes; however, when subjects gazing at the mouth, there is no significant difference between the upright face and the inverted face (*t*(34) = 2.00, *p* = 0.054, d = 0.242). In addition, these results also suggested that the weakening of the perceptual field of face by inversion was mainly reflected in the reduction of the perceptual field centered on the eyes.

## General discussion

In this study, the composite-face paradigm and the perceptual field paradigm were used to investigate the extent of involvement of the upper-half face and the lower-half face in holistic face processing. Results first showed that the composite-face effect was stronger when the participants fixated at the upper-half face relative to the lower-half face. Second, the perception field was larger when participants fixated at the eyes than the mouth. Third, inversion greatly decreased the range of perception field, when participants fixated at the eyes.

We first observed that the composite-face effect was stronger when the participants judged the upper facial half than the lower half. Two previous studies measured the composite-face effect of upper and lower faces. Although they did not directly compare the sizes of the two effects, the values of the effects in both studies were greater in the upper face than in the lower face (d′: 0.66 vs 0.35^32^; ACC: 4.9% vs 2.2%^29^, we got the values by a software named “GetData”). These results suggest that when people fixate at the upper-half face, the range of holistic face processing is relatively larger which indicates more information is included in the holistic face processing, or the connection between the upper half and the lower half is closer. In the current study, the sizes of the target area and the ignored area are same. The size relationship between the target area and the ignored area also affects the holistic face processing. Developing the Vanderbilt Holistic Face Processing Test (improved composite face task), Richler, Floyd and Gauthier varied the proportion of the composite face that that is task-irrelevant and found that the order of target segment in terms of holistic processing effect from smallest to largest was bottom 2/3, top 2/3, bottom 1/2, top 1/2, top 1/3, bottom 1/3, mouth, eyes, and nose^33^. This finding suggests that not only the fixed area, but also the size relationship between the target area, the ignored area may influence holistic face processing. As a result, it's crucial to maintain the target area's size throughout the experiment when we compared different facial areas. Future studies may need to pay closer attention to how region location and size affect the holistic face processing.

Second, results showed that the perception field was larger when the participants fixated at the eyes than the mouth on the upright face. These results have two implications. Firstly, the results that when participants fixated at the upper-half face, the range of holistic face processing was larger was consistent with the perceptual field paradigm and the composite-face paradigm. This consistency indicates the stability of these findings across experimental paradigms and could better rule out the potential influence of the tasks themselves. Secondly, as discussed earlier, the perceptual field paradigm does not use accuracy as the dependent variable, which eliminates participants’ motivation of pursuing high performance and usage of cognitive strategies during the experiment. Thus, the consistency of two experiments (cross-paradigm) does not only show that the results are stable (reliable), but also shows that the results do not seem to be interfered by cognitive strategies and should reflect the nature of the face processing accurately (valid).

The third finding of this study is that when faces were inverted, the perception field was significantly reduced. Moreover, the perception fields were reduced to similar sizes when participants fixated at the eyes and mouth. This result is consistent with previous results. Van Belle et al. also found that participants chose the center face rather than the peripheral face when they fixated at the eyes of the inverted combined face^24^, suggesting that the inversion would reduce the range of perceptual field. Van Belle and colleagues, however, did not compare the perceptual field ranges when participants fixated at different places; instead, they only examined the perceptual field when participants fixated at the eyes’ area but did not the mouth’s area. Our study compared the perceptual fields of eye and mouth areas, and found the perceptual field is larger for the eyes relative to the mouth. Also, for inverted faces, the perceptual field for eyes were reduced dramatically whereas that for mouth remained almost unchanged. In short, our finding indicated the regional specificity of perceptual fields. In addition, some studies have found that the left eye may play a greater role and be more specific than the right eye in face recognition^14,15,34^. In particular, the left eye was shown as the earliest diagnostic feature from the eye movement data^14^. Indeed, face processing ability, according to Royer et al., is associated with a systematic increase in the use of the eye area, particularly the left eye from the observer's perspective^34^. Even though the current study did not find a difference in the holistic processing range between the left and right eyes, the results above suggest that there may be additional differences between the two eyes, which we can use eye movement tracking to further examine in future research.

The theoretical implications of above results can explain two seemingly contradictory experimental results in previous studies on the performance of eye recognition and the holistic face processing. On the one hand, the processing of eyes' area is deeply involved in face processing. For instance, eyes (relative to mouths) provided more diagnostic information for face recognition^14,15,35,36^. Eyes themselves as independent features have higher priority and are more important in face perception^15,35–37^. Damages to the processing of the eyes' area of a face also severely weaken holistic face processing^38,39^. On the other hand, it seems that the processing of eyes' area was not closely related to holistic face processing. For example, inversion of face would seriously impair people’s ability to recognize faces^2,36,37^, but it did little damage to their ability to recognize eyes (by contrast, the performance of recognizing mouth had a strong inversion effect)^40–43^. The superficial contradiction between these two lines of evidence could be explained by the differences in the range of perceptual fields. First, people’s perception of eyes' area is stable; however, holistic face processing does not only process the eyes' area, but also integrated the information within and outsides the eyes' area. The larger perceptual field (covering nose, mouth, etc.) means stronger integration. Face inversion would further weaken this integration, reducing the perceptual field to just the range around the eyes. The processing of the eyes is still "anti-inversion," but the processing of the mouth area in inverted faces is severely damaged because its perception field does not cover the mouth's area. As a result, inversion will harm the holistic face processing, but the processing of the eyes will still be unaffected. In brief, people make high-speed and accurate judgments of face identity, which requires the integration of whole-face information. In the process of integration of the whole-face information, eyes play the role of "key part"^44–46^. Importantly, restricting the processing of eyes will harm holistic face processing, but the damage of holistic face processing does not necessarily harm the processing of eyes.

Further, we try to make some theoretical speculations on the specific process of how holistic face processing integrates face information and review some experimental evidence. Firstly, in space, the integration of face information may have default central vs. peripheral regions. The central region covers the upper facial half by default, so the upper facial half is more important relative to the lower facial half. Secondly, in terms of time course, the central region is first processed, and the process is faster, while the peripheral region is later processed and the process is slower. Thirdly, in terms of the integration mechanism, the upper and lower halves of a face are mutually spliced in physical space, which is mapped to holistic face representation. Fourthly, this splicing is hierarchical: the basic level is the integration of within-region information (the eyes or the upper half), and the higher level is the integration of between-region or whole-face information. These two levels together constitute the organizational mechanism for holistic face processing. The evidence supporting above theoretical speculation comes from research on early processing of faces. For example, the visual search task found that the eyes had a very high priority in visual search^16,47^; eye movement studies found that when people looked at faces, the probability of the first fixation on the eye area (including the eyes themselves and surrounding areas) was extremely high^15,48^, and 1–2 gaze points could allow participants to recognize faces quite accurately^15^; ERP studies have found that the amplitude of N170 (which is considered to be the specific component for holistic face processing)induced by eyes was much higher than other features on the face^19,20,49–51^. These results suggest that the human cognitive nervous system may have an "eye detector" that allows people to accurately detect the eyes at the very early stage of perception, completing the first stage of face perception. After detecting the eyes, face perception enters the second stage. The face perception system gradually constructs a whole representation of the face with the eyes being the central area. To this time point, the default template for whole-face representation comes into play. This default template is upright, so the information integration is relatively smooth for upright faces, but not for inverted face, which is manifested as the face inversion effect in the experimental results. In summary, we call the speculation above as the "regional hierarchical integration hypothesis" for holistic face processing. In the current study, we adopt the perspective of a default template for whole-face representation, and thought the holistic processing is specific to faces. Faces are represented as gestalts on which individual face components are adhered to form a larger "face template"^52^. Face-specific recognition ability, the face-inversion effect, and the composite-face effect were found to be more correlated in monozygotic twins than dizygotic twins, which provides significant evidence to the face specificity hypothesis^53^. The congruency effects, however, do not just occur in faces. Richler et al. had shown that contextually induced congruency effects can occur within a single trial between objects of different categories^54^. Specifically, trials that contained aligned faces led to congruency effects for Greebles (a kind of artificial object). This result is difficult to explain by the idea of a face template. Richer et al. suggested a kind of generalized expert knowledge works in such conditions^54^. When participants become experts at individuating objects from a novel category, they show a congruency effect that is modulated by alignment, just as is found with faces. The role of context, especially the transfer of congruence effects across different categories of objects, suggests that the holistic processing of faces is very complex.

There are several different composite-face paradigms^55–57^. The standard design (or original design, partial design) and the complete design are the most widely used paradigms^55–58^. There has been an intensive debate between both paradigms^59–61^. In this study, we chose the standard paradigm to study the effects that are specific to upright faces. It should be noted that the complete paradigm measures the “attentional interference”, and it produces comparable composite effects for both upright and inverted faces^62^, as well as other non-face objects^63^. In other words, the complete composite paradigm does not seem to measure the upright specific mechanism that we are interested in this study. Therefore, we employed the standard composite face task. Considering that holistic processing measured by the standard composite task is likely confounded by response bias^62^, we additionally used the perceptual field paradigm in Experiment 2 to avoid the defect in the standard composite tasks. The perceptual field paradigm employed a two-alternative-force-choice task, rather than a same/different judgement, whereby the response bias, if there is any, would not confound the behavioral performance. The results in Experiment 2 replicated those in Experiment 1; that is, the range of the holistic processing or peripheral processing were larger when participants looked at the upper halves of the faces compared to the lower halves of the faces. The consistency in results of both experiments exclude the possibility that the results in Experiment 1 was distorted by response bias. Some researchers suggested that in the standard paradigm, the congruency and response type are confounded, and the alignment effects may arise from differential response biases. A preprinted study by Jin et al. offered an alternative explanation of difference between standard design and complete design^55^. The researchers found that there are two aspects of the influence of irrelevant facial parts on the target parts in the complete composite paradigm: facilitation in congruent trials and interference in incongruent trials. And they varied from each other. Consistent with our findings, interference was only observed for identification of the top halves. But both upper and lower halves could facilitate the identification of the other parts. According to this study, the difference between the results of the standard paradigm and the complete paradigm may not be due to reaction bias, but rather to the distinct holistic processing components included in each paradigm. Future research can investigate whether there are differences between upper and lower halves in the congruent and incongruent conditions using the complete paradigm, and whether these differences are related to the holistic processing observed in other paradigms, such as the perceptual field paradigm used in this study.

The participants in Experiment 1 and 2 were from different samples. Critically, similar results were observed in both experiments: larger range of holistic processing for upper compared to lower facial halves. The consistency in results strengthens the evidence and suggest the universality of the findings. However, with different participants, we were unable to explore the relationships between these two effects. Future studies may recruit one group of participants to complete both tasks to investigate how the differences in the standard composite effect between upper and lower halves related to those in perceptual field paradigms via the individual difference approach.

To test the "regional hierarchical integration hypothesis", future research can no longer be limited to the contrast between the eyes' area and the mouth's area, but can also include other regions and face components, such as the nose’s area. The upper facial half is assumed to be the default central region, within which not only does eyes contribute the information processing, but eyebrows also play an important role^64,65^, implying that the integration processing of the eyes and the eyebrows may have important influences on holistic face processing. Then, extending from the central region to the peripheral region, the rule of information integrating between the eyes and the nose, or the mouth is also worth noting. In order to fully comprehend the mechanics underpinning face information integration, future study should investigate into more local face regions as well as the rule of information integration within and between regions.

## Data Availability

The data collected and analyzed during the current study are available from the corresponding author upon request.
